# Edible Mushrooms as Functional Ingredients for Development of Healthier and More Sustainable Muscle Foods: A Flexitarian Approach

**DOI:** 10.3390/molecules26092463

**Published:** 2021-04-23

**Authors:** Arun K. Das, Pramod K. Nanda, Premanshu Dandapat, Samiran Bandyopadhyay, Patricia Gullón, Gopalan Krishnan Sivaraman, David Julian McClements, Beatriz Gullón, José M. Lorenzo

**Affiliations:** 1 Eastern Regional Station, ICAR-Indian Veterinary Research Institute, 37 Belgachia Road, Kolkata 700 037, India; npk700@gmail.com (P.K.N.); pdandapat@gmail.com (P.D.); samiranvet@gmail.com (S.B.); 2 Nutrition and Bromatology Group, Department of Analytical and Food Chemistry, Facultad de Ciencias de Ourense, Universidad de Vigo, 32004 Ourense, Spain; pgullon@uvigo.es; 3 Central Institute of Fisheries Technology, Willingdon Island, Cochin, Kerala 682 029, India; gkshivraman@gmail.com; 4 Department of Food Science, University of Massachusetts, Amherst, MA 01060, USA; mcclemen@umass.edu; 5 Department of Chemical Engineering, Faculty of Science, Campus Ourense, University of Vigo, As Lagoas, 32004 Ourense, Spain; bgullon@uvigo.es; 6 Centro Tecnológico de la Carne de Galicia, Adva. Galicia n° 4, Parque Tecnológico de Galicia, San Cibrao das Viñas, 32900 Ourense, Spain; 7 Área de Tecnología de los Alimentos, Facultad de Ciencias de Ourense, Universidad de Vigo, 32004 Ourense, Spain

**Keywords:** mushrooms, bioactive compounds, functional ingredients, meat, fish, quality aspects, functional muscle foods

## Abstract

Consumers are increasingly interested in nutritious, safe and healthy muscle food products with reduced salt and fat that benefit their well-being. Hence, food processors are constantly in search of natural bioactive ingredients that offer health benefits beyond their nutritive values without affecting the quality of the products. Mushrooms are considered as next-generation healthy food components. Owing to their low content of fat, high-quality proteins, dietary fibre and the presence of nutraceuticals, they are ideally preferred in formulation of low-caloric functional foods. There is a growing trend to fortify muscle food with edible mushrooms to harness their goodness in terms of nutritive, bioactive and therapeutic values. The incorporation of mushrooms in muscle foods assumes significance, as it is favourably accepted by consumers because of its fibrous structure that mimics the texture with meat analogues offering unique taste and umami flavour. This review outlines the current knowledge in the literature about the nutritional richness, functional bioactive compounds and medicinal values of mushrooms offering various health benefits. Furthermore, the effects of functional ingredients of mushrooms in improving the quality and sensory attributes of nutritionally superior and next-generation healthier muscle food products are also highlighted in this paper.

## 1. Introduction

Muscle foods, such as meat and fish, play an important role in the daily diet of most consumers due to their desirable sensorial attributes and beneficial nutritional properties, including high levels of good quality proteins, vitamins, and minerals. However, muscle foods are deficient in vitamin C, calcium, dietary fibre, and antioxidants [1]. Moreover, consumption of processed food has been linked to certain chronic health problems, such as an increase in diabetes and obesity [2,3]. The increased awareness of consumers about the possible links between diet and health is leading to shifts in their dietary patterns towards healthier food products. Healthier eating habits include reducing the consumption of ingredients that may cause health problems such as saturated fat, sugar, and salt, and increasing the consumption of ingredients that may promote human health such as unsaturated fatty acids, vitamins, minerals, and nutraceuticals [4]. Adopting these dietary habits favours the maintenance of a healthy weight, as well as minimizing the risk of some lifestyle diseases [5]. As a result, there is increasing demand for healthier food products that consumers can easily incorporate into their diets. Moreover, the raising of animals to produce muscle foods is undesirable from an environmental viewpoint, since it leads to more greenhouse gas emissions, land use, water use, and pollution than growing arable crops [6]. Consequently, it is also desirable to reduce the total amount of animal foods within the human diet. This can be achieved by avoiding animal products altogether (vegan), avoiding meat products (vegetarian), or reducing the amount of meat products in the diet (flexitarian). This latter approach is suitable for those who want to adopt a healthier and more sustainable diet, but still want to consume some meat.

As a result of these concerns, the food industry is reformulating existing products and creating new products to make them healthier and more sustainable [7,8]. In this article, we focus on the creation of foods designed for the flexitarian market. Specifically, we focus on replacing part of meat or fish products with healthy and more sustainable natural ingredients: mushrooms. Edible mushrooms are considered to be healthy food ingredients because they contain high levels of quality proteins, dietary fibres, vitamins, minerals, and phenolic compounds [9,10,11,12]. Moreover, they have a relatively low concentration of fat and digestible carbohydrates, which makes them suitable for improving the nutritional profile of foods [13]. Some mushrooms have also been reported to contain constituents that exhibit beneficial therapeutic effects [14]. For instance, polysaccharide-protein complexes and lectins have been reported to have immunomodulatory and antitumor activities [15,16], hypotensive effects [17], and anti-angiogenesis effects [18]. There is, therefore, growing interest in incorporating mushrooms into muscle foods, thereby reducing the proportion of meat present [19,20]. One of the advantages of using mushrooms for this purpose is that they have good compatibility with meat products because of their umami flavour and fibrous meat-like texture [21,22,23,24].

This review outlines the utilization of the edible parts of mushrooms as functional ingredients in muscle food products, such as meat and fish. In particular, the impact of mushrooms on the nutritional and quality attributes of these products are critically reviewed, including their physicochemical properties, microbiological stability, chemical stability, and sensory aspects.

## 2. Mushroom—A Culinary Delicacy

Mushrooms are valued around the word as culinary delicacies and are popularly known as “vegetable meat” in many cultures. Botanically, they are the fruiting bodies of macroscopic filamentous saprophytic fungi that grow above ground. Their beneficial effects on human health and nutrition were recognized in early Greek, Egyptian, Roman, and Chinese civilizations [25,26,27]. Mushrooms can be conveniently categorized into three major groups according to their applications edible (54%), medicinal (38%), and wild (8%) [28]. It has been estimated that there are at least 12,000 mushroom species worldwide, with around 2000 of them being suitable for edible and/or medicinal application, but only 35 being currently cultivated commercially [29]. Nutritionally, mushrooms have many positive benefits for the human diet: they are low in fat, high in protein, and high in dietary fibre, as well as being good sources of vitamins, minerals, and nutraceuticals [30,31]. As a result, the global mushroom market has grown considerably over the past few years with 34 billion kg production and per capita consumption exceeding 4.7 kg in 2013 [28]. Indeed, mushroom production is currently a multibillion-dollar industry with an annual turnover of around USD 35 billion in 2015 and estimated to exceed USD 59 billion in 2021, growing at around 9.2% from 2016 to 2021 (http://www.zionmarketresearch.com/report/mushroom-market, accessed on 8 January 2021). Commercially, mushrooms are mainly cultivated on agricultural residues, which enables these waste materials to be converted into a valuable human food source [32], while also reducing waste and environmental pollution.

Some of the most important commercially cultivated mushrooms are *Agaricus bisporus* (agaric or button), *Lentinula edodes* (shiitake), *Flammulina velutipes* (enoki or winter mushroom), *Pleurotus eryngii* (king trumpet mushroom), *Pleurotus ostreatus* (oyster mushroom), *Volvariella volvacea* (paddy straw mushroom), *Calocybe indica* (milky mushroom), *Hericium erinaceus* (pom pom or lion’s mane mushroom), *Boletus edulis* (porcini, cèpe, or king bolete mushroom), *Grifola frondosa* (maitake or hen of the woods mushroom), and *Agrocybe aegerita* (pioppini) [33]. Notably, about 85% of the world’s cultivated edible mushrooms is represented by only five genera viz. *Lentinula*, *Agaricus*, *Pleurotus*, *Auricularia* and *Flammulina* [28], despite the fact that a wide variety of other edible mushrooms could also be cultivated commercially on a large scale. There are some extrinsic and intrinsic factors that influence the stalk height, stalk diameter and cap size in cultivated mushroom. The most important factors responsible for increased production of cultivated edible mushroom are temperature, humidity, fresh air, and compact material. 

## 3. Mushroom as Bioactive Functional Food Ingredients

Although many ingredients are used while preparing processed food products, the role of food ingredients that merits special mention are those with inherent nutritional as well as functional properties that influence the quality of finished food products. Therefore, ingredients are now considered as an essential part and parcel of any food product development process. However, the ingredients or compounds obtained from natural sources and generally regarded as safe are of great interest because of their safety and health characteristics [34]. As per the Food and Drug Administration, these are the substances that influence various attributes and properties of any food, either directly or indirectly. They are included at any down streaming stage of processing, be it production, packaging or storage of food, till it reaches the consumer. The purpose is not only to improve nutritional quality and safety but also the freshness, appearance, and overall acceptability of the food products by modifying taste and texture. These additives are often considered as nutraceuticals when these or part of their components exert medical or positive health benefits and play a vital role in the prevention and treatment of various diseases [35,36]. The nutraceuticals could be either whole food or its part, or even a single component or extract of food which is regularly being used as a dietary supplement. A food item is termed as “Functional” only when these nutraceuticals are incorporated in the food or its formulation to achieve specific target function such as improving the well-being as well as quality of human life by reducing the risk of disease beyond its nutritional value [35,37]. 

Mushrooms, which belong to filamentous higher fungi, are known for their nutritional richness, low caloric value, taste, and nutraceutical properties. Due to their unique nutritional as well as textural properties, they are used as a dietary supplement and often considered as an alternative source of meat, fish, vegetables, fruits, etc. [38]. Moreover, mushrooms are a source of high-quality protein produced in huge quantity from recycling worthless agro-wastes including agro-industrial waste per unit area and time [39,40]. Therefore enriching or fortifying diets or food products with such a good source of protein containing all the essential amino acids may help in reducing the incidences of protein-energy malnutrition in humans [41]. Furthermore, owing to the presence of numerous secondary metabolites or nutraceuticals or biologically active compounds having medicinal value, mushrooms can also be used as bio-therapeutic agents [42,43].

Generally, mushrooms possess all three functionalities of food—nutrition, taste, and physiological functionalities. Mushrooms have a peculiarly pleasant savory taste called umami due to presence of sodium salts of free amino acids such as glutamic and aspartic amino acids and 5′-nucleotides [44]. The umami taste, also called the palatable taste, is nothing but the overall food flavour enhanced by mono-sodium glutamate [45]. Hence, mushrooms are preferable and adaptable in most food formulations due to this unique flavour.

Again, the umami taste peptides and umami-enhancing peptides are also considered to be important components which influence the sensory quality of mushroom. Peptides with different structures and length possess unique taste properties including sweet, bitter, umami, sour and salty. They are usually tasteless in water, but they can increase the salty, sweet, sour, bitter or umami taste in combination with corresponding tastants [46]. Various researchers have reported that some dipeptides or tripeptides containing Glu such as Glu-Glu, Glu-Asp, Glu-Asp-Glu, Glu-Gly-Ser enhance umami taste [47]. Recently umami peptides (2 tripeptides and 3 dipeptides) were isolated from hydrolysates of dried shiitake mushroom and these peptides are believed to be responsible for specific taste of shiitake mushroom. They also contribute to the unique taste of mushrooms or even interact with other volatile compounds to influence the whole flavour of foods [48]. In an another study, umami taste peptides like Gly-Leu-Pro-Asp and Gly-His-Gly-Asp isolated from the mushroom *Agaricus bisporus* are reported to act as key molecules for kokumi taste [49]. Kokumi taste is best described as flavor characteristics such as mouthfulness, complexity, and continuity. Kokumi taste substances have slight taste or even no taste by themselves, but they can enhance the flavor of the basic tastes, such as sweet, salty, and umami [50]. Interestingly, when incorporated to a blank chicken broth, these peptides from *Agaricus bisporus* can elicit new taste sensations, such as mouthfulness and complexity [49].

### 3.1. Nutritive Profile of Mushrooms

As stated earlier, mushrooms are excellent sources of dietary fibre and rich in protein possessing all nine amino acids that are essential for humans [26] but low in fat and calories [13]. In general, the mushroom fruit bodies contain 5–15% dry matter, 19–35% protein and low fat content (Table 1). Indeed, the protein content of mushrooms is almost four times greater than tomatoes and carrots, six times greater than oranges, and 12 times greater than apples [40,51]. Mushrooms, both pilei and stems are excellent source of dietary fibre mainly due to the presence of non-starch polysaccharides. Stems of mushroom are mainly composed of insoluble dietary fibre (IDF) and glucans. Hence, mushroom stem could be utilized for preparation of biologically active polysaccharide complexes as food supplement [52]. In a recent study, stem of enoki or winter mushroom (*Flammulina velutipes*) reported to contain 32% dietary fibre [53]. Again, the low fat and high-fibre content of mushrooms may help in preventing hypertension and hypercholesterolemia, as well as being beneficial in weight control [54]. Mushrooms are also healthy sources of essential fatty acids (52–87% unsaturated fatty acids), mostly in the form of linoleic acid, which cannot be directly synthesized in the human body but is required for health [55]. Mushrooms are also rich in indigestible carbohydrates, which makes them promising sources of novel prebiotic components [56,57]. The low glycemic index and high mannitol content of mushrooms is also believed to be beneficial for diabetics [58].

Being an excellent source of dietary fibres and proteins, mushrooms also have a low sodium content, and contain a diverse range of micronutrients, such as vitamins B_1_, B_2_, B_12_, C, D, E, niacin, and folate [59]. Despite being cultivated in the dark and being a non-animal source of food, mushrooms contain significant levels of vitamin D, which is often referred to as “the sunshine vitamin”. Upon exposure to sunlight or ultraviolet (UV)-B light, the vitamin D (specially vitamin D_2_) content of mushrooms increases appreciably, which can play a significant role in the bone and cartilage health of vegans and vegetarians [60,61,62].

Furthermore, mushrooms are a rich source of essential minerals like iron, copper, manganese, and zinc that play an important role in the proper functioning of different metabolic pathways. Indeed, the levels of some important trace elements (such as potassium and phosphorus) are typically considerably higher in mushrooms than in most vegetables [63]. Edible mushrooms can also produce a range of flavonoids, which may exhibit health benefits [64].

It should be noted that the nutritional composition of mushrooms varies considerably depending on factors such as species, intra-species genetic variability, maturity, growth conditions, geographic location, environmental conditions, and post-harvest conditions [65,66]. The chemical composition of some popular varieties of mushroom reported by different researchers, are presented in Table 1.

**Table 1:** Chemical composition of some common and popular mushroom species.

Mushroom	Common Name	Protein	Fat	Crude Fibre	Ash	Carbohydrate	Energy Value (kcal/100 g)	Ref.
Fresh/Raw (g/100 g)								
*Agaricus bisporus*	White button, Agaric, Pizza	3.00	0.34	1.45	0.79	3.69	24	[27]
*Flammulina velutipes*	Winter, Enoki	2.66	0.28	2.80	0.91	8.42	29	
*Grifola frondosa*	Maitake	1.94	0.20	2.70	0.52	2.70	29	
*Pleurotus ostreatus*	Oyster	2.00	0.99	2.10	0.24	5.35	39	[67]
*Pleurotus sajor caju*	Oyster	23.3	3.0	35.6	3.2	65.5		[68]
Dried (g/100 g)								
*Pleurotus eryngii*	King trumpet oyster	28.8	3.0	-	3.5	52.2	-	[69]
*F. velutipes*	Winter, Enoki	18.42	2.94	7.81	6.33	56.37	-	[70]
*Termitomyces heimii*	Wild edible	23.75	3.58		4.40	54.70	345	[54]
*A. bisporus*	White button, Agaric, Pizza	29.29	2.22	24.56	7.12	20.57	-	[71]
*P. sajor caju* (stalk)	Oyster	22.51	2.6	16.24	8.54	40.2	-	[41]
*P. sajor caju* (cap)	Oyster	26.34	3.07	8.97	10.37	38.17	-	
*P. ostreatus*	Oyster	20.04	8.65	-	7.78	60.21	421	[67]
*Tricholoma nauseosum*	Matsutake	18.1	2.0	30.1		31.1	-	[55]
*Sarcodon imbricatus*	Scaly hedgehog	12.0	2.8	5.1		64.6	-	
*G. frondosa*	Maitake	21.1	3.1	10.1	7.0	58.8	-	[72]
*Hericium erinaceus*	Pom pom or Lion’s mane	22.3	3.5	7.8	9.4	57.0	-	
*Boletus aereus*	Bronze bolete or The dark cep	17.86	4.4	-	8.87	72.83	306	[73]
*Boletus edulis*	Cep or Porcini	21.07	2.45	-	5.53	70.95	423	
*Boletus reticulatus*	Summer cep	22.57	2.55	-	19.72	55.16	297	
*Pleurotus florida*	Oyster	34.56	2.11	11.41	7.40	31.59	-	[74]
*Pleurotus ostreatus*	Oyster mushroom	30.92	1.68	12.10	7.05	31.40	-	
*Calocybe gambosa*	St. George or Milky	15.46	0.83		13.89	69.82	317	[75]
*Clitocybe odora*	Aniseed	17.33	2.46		9.55	70.66	431	
*Coprinus comatus*	Shaggy ink cap	15.67	1.13		12.85	70.35	525	
*F. velutipes* (stem waste)	Winter, Enoki	13.50	1.47	32.30	8.24	63.89	-	[53]
*P. florida*	Oyster	27.83	1.54	23.18	9.41	32.08	-	[76]
*Russula delica*	Milk-white	26.25	5.38	15.42	17.92	34.88	-	
*Lyophyllum decastes*	Fried chicken	18.31	2.14	29.02	14.20	34.36	-	
*Fistulina hepatica*	Beefsteak fungus	63.69	2.63	-	11.30	22.98	364	[77]
*Laccaria laccata*	Deceiver or Waxy laccaria	62.78	3.76	-	20.69	12.77	336	
*Suillus mediterraneesis*	-	24.32	2.61	-	27.64	45.42	302	
*Tricholoma imbricatum*	Matsutake	50.45	1.88	-	6.45	41.21	383	
*Volvariella volvacea*	Paddy straw	29.5	5.7	-	10.4	60.0	374	[78]
*Lentinula edodes*	Shiitake	17.5	8.0	-	8.0	67.5	387	
*Auricularia polytricha*	Wood ear, Jelly ear	7.7	0.8	-	14.0	87.6	347	
*Tremella fuciformis*	White Jelly	4.6	0.2	1.4	0.4	94.8	-	
*Pholiota microspore*	Nameko	20.8	4.2	-	6.3	66.7	372	
*Calvatia utriformis*	Mosaic puffball	20.37	1.90	-	17.81	59.92	744	[60]
*Lycoperdon echinatum*	Spiny puffball	23.52	1.22	-	9.43	65.83	544	
*Russula cyanoxantha*	Charcoal burner	16.80	1.52	-	7.03	74.65	590	
*Agaricus campestris*	Field or Meadow	18.57	1.1		23.16	58.16	-	[79]
*Boletus armeniacus*	-	18.25	1.56		12.09	68.10	-	
*Tricholoma giganteum*	Matsutake	16.1	4.3	4.5	5.0	70.1	-	[80]
*V. volvacea*	Paddy straw	30.1	6.4	11.9	12.6	50.90	-	

### 3.2. Nutraceutical Components in Mushrooms

Mushroom nutraceuticals are natural compounds found in mushrooms that may have health benefits by reducing the risks of certain diseases or by improving human performance [42,81,82]. The potential health-promoting and disease-preventing effects of mushroom nutraceuticals have been attributed to a broad range of biological activities, which are discussed in this section. 

Mushrooms have been reported to contain different kinds of nutraceuticals, including lectins, triterpenoids, ganoderic acid, β-glucan, phenolics, flavonoids, hispolon, calcaelin, proteoglycan, lentinan, laccase, nucleosides, nucleotides, and ergosterol [31,83,84,85]. As far as polyphenolic compounds, are concerned fruiting bodies of mushrooms as well as mushroom extracts contain significant amounts of phenolic acids, especially derivatives of benzoic acid and derivatives of cinnamic acid. Different mushroom species have been found to contain protocatechuic, p-hydroxybenzoic, vanillic, salicylic, p-coumaric, gallic, gentisic, syringic, veratric, cinnamic, caffeic, and ferulic acids [86]. The biological activities and potential health benefits of some of these nutraceuticals have been extensively studied. For example, a number of polysaccharides found in mushrooms, including chitin, β-glucan, α-glucan, mannans, xylans and galactans, have been reported to have potential health benefits [43,61,87]. In general, the nutraceuticals in mushrooms may exhibit a broad spectrum of different biological activities depending on their chemical structure and their interactions with biochemical processes, including anti-inflammatory, anticarcinogenic, antitumor, antimutagenic, antidiabetic, antibacterial, antiviral, anti-obesity, and anti-hypercholesterolemic activities [36,60,88,89]. As their application in promoting human health have been extensively reviewed by many previous researchers [42,43,55,66,82,84,90], we do not consider them further in this review.

### 3.3. Prebiotic Effects of Mushrooms 

There is growing evidence that human health can be promoted by consuming a diet that establishes a diverse microbiome in the colon [91]. In particular, diets that favour the growth of beneficial bacteria such as *Lactobacillus* and *Bifidobacterium*, while suppressing the growth of detrimental bacteria such as *Clostridia* and *Bacteroides*, may promote health [56,91]. Prebiotics are non-digestible and fermentable food components, such as oligosaccharides, dietary fibres, and non-digestible starches, that promote health by selectively modulating the composition and/or activity in the intestinal microbiota [61,92,93]. Mushrooms have been reported to contain numerous constituents that exhibit prebiotic activities, including chitin, hemicellulose, β-glucan, α-glucan, mannans, xylans, and galactans [57]. Some of the important mushroom species that have been reported to exhibit strong prebiotic activity include *L. edodes* (Shiitake), *Trametes versicolor* (Yunzhi), and *Ganoderma lucidum* (Reishi). 

In a study, Chou et al. [94] reported that prebiotics (polysaccharides and protein-polysaccharide complexes) from mushrooms passed through the human stomach and small intestine without digestion, then reached the colon where they stimulated the growth of healthy bacteria (*Lactobaccilus acidophilus and Bifidobacterium longum* subsp.). Similarly, glucans from *P. ostreatus* and *P. eryngii* [95] and *G. lucidum* [90] have also been shown to stimulate the growth of *Bifidobacterium* sp. and *Lactobacillus* sp. 

**Figure 1:**
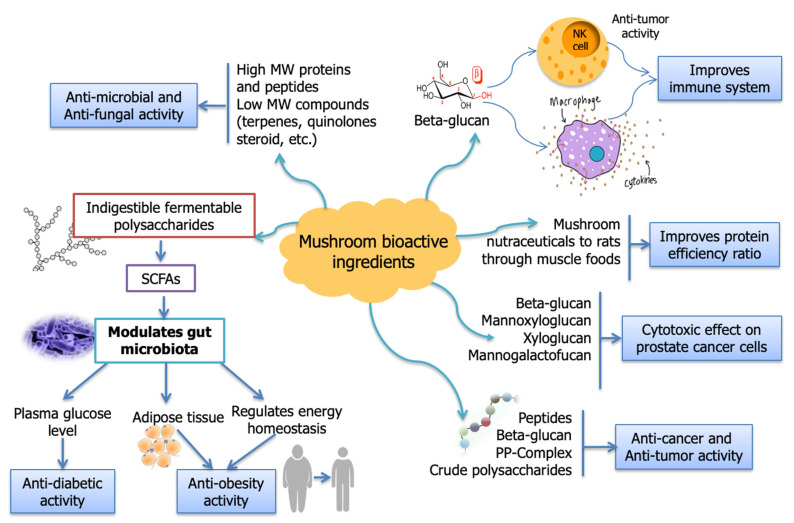
Schematic diagram depicting health promoting and medicinal properties of mushroom bioactive ingredients (MW = Molecular weight; NK cell-Natural killer cell; PP-complex: Protein-polysaccharide complex; SCFA = Short-chain fatty acids).

Prebiotic mushroom polysaccharides are also reported to exhibit antiobesity and antidiabetic effects by regulating the energy homeostasis and plasma glucose levels of the host [61]. The restoration of energy balance is believed to be due to the supply of alternate energy sources from short-chain fatty acids produced during fermentation of the non-digestible carbohydrates in the colon [96]. Some *in vitro* studies have also reported that extracts from *G. lucidum* can modulate the gut microbiota in a manner that may help prevent obesity [97,98]. Other studies have reported that the polysaccharides from various mushroom varieties may be able to ameliorate metabolic syndromes (including diabetes), such as *Agaricus brasiliensis*, *Agrocybe chaxingu*, *Catathelasma ventricosum*, *Pleurotus abalonus*, *Tremella fuciformis*, *G. frondosa*, *and G. lucidum* [61,99]. The potential health promoting and medicinal properties of various bioactive ingredients found in mushrooms are summarized in Figure 1.

## 4. Effects of Edible Mushrooms on Muscle Food Products

Edible mushrooms, due to richness in nutritive value and functional food components, make them an unmatched source of healthy food and are regarded as superior nutritional supplements [36,53]. To harness the goodness of nutritional, nutraceutical and other medicinal values, mushrooms are not only used directly as food but also as raw materials in formulation and development of new functional foods for health-conscious consumers. Other than these values, mushrooms are preferred as additives by the food processors due to their aroma, taste and inherent texture-modifying functional properties [100,101] which are reported to positively influence the flavour, appearance, overall acceptance and shelf-life, when incorporated in various processed food formulations [53]. 

Considering their enormous benefits, varieties of food products such as breads [102], fish and meat products [103], cookies [104], other preparations like instant soups, pasta, snack seasonings, casseroles, and rice dishes [105,106] are being formulated incorporating mushrooms as functional bioactive components that is stated to improve the nutritional profile and potential health benefits [104]. Although quite a large number of research articles are available highlighting the use of mushrooms as potential functional compounds in various food applications, this review limits its focus on the potential application of mushrooms in muscle foods (meat and fish) only. 

**Figure 2:**
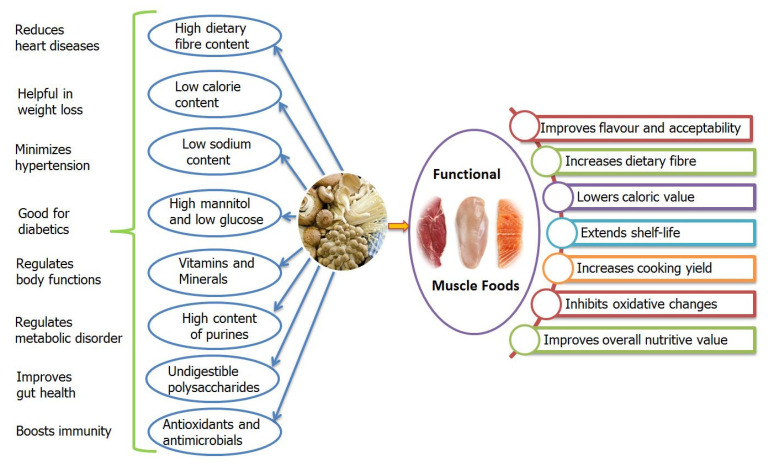
Schematic diagram showing the influence of mushroom nutrients on quality attributes of muscle foods vis-à-vis human health effects.

Incorporation of mushroom and its parts not only influences the desirable texture, taste, flavour, and stability of muscle food products considerably but also enriches them with nutritive and functional health values [36,104,107,108,109]. Figure 2 indicates the beneficial effect of mushrooms on quality attributes of muscle foods and associated health benefits. Further mushroom is better known for its low sodium content [110]. For example, the fruiting bodies of *Agaricus* sp. contain 396 mg sodium/kg [111] which is low amongst the vegetables [112]. On the other hand, processed meats contain 7–39 g sodium chloride/kg [113]. Dietary intake of such a higher amount of sodium is often linked with various diseases and increases the risk of hypertension and cardiovascular diseases [114]. Therefore, pre-mixing or blending mushrooms in processed meats may help in reducing the sodium content of the products, offering more nutritional and health benefits to consumers [115]. 

Over the years, a number of researchers have successfully incorporated mushrooms and its various parts (stipes and stem wastes) in formulation of various muscle food products like chicken sausages [70], salted cooked beef [116], tuna meat [117], kuruma shrimp [118], emulsion-type pork sausages [109], traditional Turkish meatball [108], fermented pork sausages [107], sutchi catfish patties [71] etc. A summary on the effects of edible mushrooms on physico-chemical properties, colour and oxidative stability, shelf-life and sensory attributes of muscle food products is presented in Table 2.

## 5. Effect of Mushrooms on Quality Aspects of Muscle Food Products

### 5.1. Mushrooms on the Physicochemical Properties of Muscle Foods

The quality and acceptability of muscle foods depends on a number of different physicochemical properties including chemical composition, pH, water-holding capacity (WHC), emulsion stability, and cooking yield [8,132]. The pH of muscle foods is particularly important because it influences their WHC, juiciness, cooking yield, texture, and shelf-life by regulating microbial growth [1,133]. Studies have shown that incorporation of winter mushroom (*F. velutipes*) powder into emulsion-type pork sausages increased their pH, WHC, and cooking yield by decreasing the exudation of fat and water from the sausages [109]. Similarly, Cha et al. [126] reported that the incorporation of white jelly mushroom (*T. fuciformis*) into pork patties significantly increased their oil retention and cooking yield. Moreover, the introduction of 25% fresh mushroom into chicken patties has been reported to give an improved moisture retention (77%) and cooking yield (81%) [134]. The increase in cooking yield and water/fat retention are not only beneficial from technological and sensorial, but also from an economic viewpoint. In a recent study, the inclusion of enoki mushroom (*F. velutipes*) stem wastes into goat meat nuggets was shown to give a higher pH, emulsion stability, cooking yield, and WHC [53]. Likewise, Bao et al. [135] reported a slight increase in the pH of beef and fish products after addition of enoki mushroom extracts. The observed increase in pH after mushroom addition could be due to the relative abundance of basic amino acids compared to acidic amino acids in these products [136], as well as the natural buffering capacity of the mushroom proteins [137]. In contrast, shiitake (*L. edodes*) extracts were found to reduce the pH of fermented sausages during 30 days storage at 15 °C [130], which may have been due to the presence of lactic acid bacteria that generated acids within the fermented sausages. 

The addition of mushroom also influences the chemical composition and nutritional profil of muscle foods. As discussed earlier, these effects can be attributed to the presence of relatively high levels of protein, minerals, and dietary fibres in mushrooms. The incorporation of dried mushroom (*P. ostreatus*) into beef patties has been reported to increase the protein, fat, and ash content of the end product [67]. Similarly, the incorporation of dried grey oyster mushrooms significantly decreased the fat content of cooked chicken patties [68]. The incorporation of dried enoki mushroom extracts into goat meat nuggets has been reported to increase their dietary fibre and ash contents [53]. Wan Rosli et al. [124] reported that the incorporation of oyster mushroom (*P. sajor caju*, PSC) powder into chicken meat decreased the fat content but increased the dietary fibre content in frankfurters in a dose-dependent manner. 

Taken together, these studies clearly show that the composition of muscle-based foods can be manipulated by adding different types and amounts of mushrooms during processing. Incorporation of mushrooms either in the form of powder or extract influences the physicochemical properties, sensory attributes, and nutritional profiles of muscle foods.

### 5.2. Mushrooms on Lipid Oxidation in Muscle Foods

The oxidation of the lipids and proteins in muscle foods is undesirable because it leads to rancid odours, off-flavours, discolouration of the products [138,139,140]. Again, lipid oxidation by different means produces free radicals (such as alkyl, alcoxyl, and peroxyl radicals) which have been observed to induce protein oxidation [141]. For example, cooking or heating muscle food products above 60 °C initiate oxidative cleavage of the porphyrin ring, resulting in release of heme iron, which can lead to increased lipid and protein oxidation [142,143]. These oxidative reactions are extremely complex and lead to the loss of valuable nutrients, as well as the generation of numerous types of reaction products [116,140]. For instance, the essential amino acids and fatty acids may be lost [144], whereas volatile off-flavours are produced [145,146]. Many factors influence the lipid and protein oxidation, including oxygen, temperature, light, and transition metal ions. Researchers are increasingly trying to identify and utilize natural antioxidants to inhibit lipid and protein oxidation in muscle foods [147,148,149,150]. The utilization of these antioxidants helps in improving the food quality, shelf life, and nutritional profile [8,151,152].

Mushrooms contain a wide range of natural antioxidants, including phenolic compounds, ergothioneine, ascorbic acid, tocopherols, and carotenoids [36,64,153,154]. The fruit bodies and mycelium of mushrooms also contain various types of natural antioxidants, including glycosides, polysaccharides, selenium, ascorbic acid, tocopherols, and carotenoids [58]. Researchers have reported that phenolic compounds (3–11 mg/g) and flavonoids (2.5–4.8 mg/g) are the major bioactive compounds responsible for the antioxidative activity of the fruit bodies of edible mushrooms [155]. Winter mushrooms (*F. velutipes*) are also known to have strong antioxidant activity because they contain phenolic compounds such as quercetin, chlorogenic acid, gallic acid, proto-catechuic acid, and flavonoids [53,109,156]. Methanol extracts of *B. edulis* have also been reported to contain constituents that are known antioxidants, such as ascorbic acid (18.7 mg/g dw), tocopherols (18.7 mg/g dw) and phenolic acids (9.74 mg/kg dw) [73]. Waste materials (stipes) from shiitake (*L. edodes*) mushroom have also been reported to contain various kinds of natural antioxidants [157,158]. Acetone and methanol extracts of various mushrooms, including *Amanita rubescens*, *Lepista nuda*, *Cantharellus cibarius*, *Hypsizigus marmoreus*, *Lactarius piperatus*, *Polyporus squamosus*, *Mucor circinelloides*, *Russula cyanoxantha A. bisporus*, *L. edodes*, and *V. volvacea* have also been reported to exhibit strong antioxidant activity, which is mainly attributed to their high levels of phenolics and flavonoids [159,160,161,162]. 

Mushroom extracts from the fruiting bodies of edible mushrooms (*F. velutipes*) have been reported to inhibit lipid and protein oxidation in raw beef and fish (bigeye tuna) during storage, which was attributed to the presence of natural antioxidants [103,135]. Alnoumani et al. [116] incorporated dried *A. bisporus* powder into salted cooked ground beef and tested its ability to protect the lipids and proteins from oxidation during storage. The mushroom extract was found to effectively inhibit lipid and protein oxidation, with about 88–94% lower malonaldehyde values and 99% lower volatile aldehydes being produced after 16 days storage compared to the control sample. In another study, Nayak et al. [71] reported significantly lower oxidative changes in sutchi catfish (*Pangasius hypophthalmus*) patties after a button mushroom (*A. bisporus*) extract was incorporated, leading to an appreciable increase in shelf life. The antioxidative activity of mushrooms and their extracts have also been demonstrated in various other studies, including *B. edulis* extracts in beef burger patties [119], ground white mushroom in dry-fermented beef products [63], *F. velutipes* extract in bigeye tuna [117], *F. velutipes* extract in kuruma shrimp [118], and *L. edodes* extracts in fermented pork sausages [130]. 

These studies clearly show that mushrooms and their extracts contain a diverse range of natural antioxidants that can improve the quality and shelf-life of muscle food products by inhibiting lipid and protein oxidation.

### 5.3. Mushrooms on the Textural Properties of Muscle Foods

The quality attributes and acceptability of muscle foods is strongly influenced by their textural properties. Muscle foods are compositionally and structurally complex soft materials with semi-solid textures that influence their preparation, mastication, and digestion. The textural attributes of muscle foods is not only largely governed by the gel-forming and emulsification properties of the proteins they contain, but also effect by other components, such as lipids and minerals [151,163]. The incorporation of mushrooms into muscle foods influence their rheological characteristics, which must be accounted for when designing mushroom-enriched products. One of the advantages of using mushrooms in meat products is that they already have quite meat-like textures themselves because they have a very firm texture, and their dietary fibre fractions form a dense meaty texture when processed with muscle foods. As a result, they can often be incorporated up to a certain percentage into muscle foods without causing major adverse effects on their textural attributes [53,127]. Changes in quality aspects especially textural attributes of muscle foods due to incorporation of mushroom can be assessed from different studies is listed in Table 2. 

A number of researchers have examined the impact of mushrooms on the textural properties of muscle foods. Choe et al. [109] reported a decrease in hardness, springiness, gumminess, and chewiness of sausages after incorporation of a mushroom powder. Similarly, Banerjee et al. [53] reported that the hardness, springiness, cohesiveness, and gumminess of meat nuggets decreased after addition of mushroom extracts, but these effects were not statistically significant. The hardness and other textural attributes of chicken patties have been reported to decrease after replacement of 25% or 50% of chicken meat with oyster mushroom [120,134]. Decreased hardness, cohesiveness, and gumminess but increased springiness have been reported when king oyster mushroom is incorporated into surimi gel prepared from cuttlefish (*Sepia esculenta*) meat paste [121]. 

In general, these results suggest that incorporation of mushrooms into muscle foods usually leads to softening of the final products. There are a number of possible physicochemical phenomena that may account for this effect. For instance, mushrooms contain relatively high levels of dietary fibres that can form a 3D biopolymer network that traps fluids, thereby leading to a softer texture in the muscle food products [164,165]. Moreover, the incorporation of high levels of mushrooms into muscle foods reduces the concentration of solubilized muscle proteins, thereby decreasing their ability to form strong gels.

### 5.4. Mushrooms on the Appearance of Meat Products

The appearance of muscle foods, such as their opacity, colour, and surface sheen, provide a visible indication of their quality and freshness, thereby playing an important role in determining consumer purchasing decisions [166,167]. The incorporation of mushrooms into muscle foods may impact their appearance in a number of ways. Mushrooms naturally have a different colour to meat or fish and, therefore, blends will have a different appearance than meat or fish alone. Moreover, the mushroom extracts may contain particles that have sizes and shapes that are different from those present in muscle foods, which can alter their visual texture. Finally, mushrooms contain antioxidants and other molecules that may inhibit colour changes in muscle foods. In this section, we provide a brief overview of studies that have examined the impact of mushrooms on the appearance of muscle foods.

Bao et al. [135] monitored changes in the met-myoglobin concentration and colour of minced beef and bigeye tuna meat products containing winter mushroom extract during cold storage. The authors reported that the presence of the mushroom extracts significantly decreased the met-myoglobin concentration in the meat products. As a result, the desirable colour of the beef and tuna products was maintained for up to 12 and 7 days storage compared to 6 and 2 days for the control samples (no mushroom), respectively. The colour stabilizing effect of the mushroom extract was attributed to the presence of ergothioneine, which reduced the rate of met-myoglobin formation, which is known to promote discolouration in muscle foods [135,168]. Similarly, an ergothioneine extract from mushroom was shown to prolong the stability of the red colour of yellowtail and tuna fish meat during cold storage [123]. 

In another study, researchers reported that the presence of 50% oyster mushroom in cooked chicken patties did not affect their redness (a*) but did decrease their lightness (L*) and yellowness (b*) [134]. Incorporation of white winter mushroom powder into emulsion-type pork sausages was reported to have little impact on their appearance [109]. Similarly, incorporation of up to 20% *A. bisporus* mushroom into beef burgers did not strongly affect their appearance [127]. The introduction of shiitake mushroom powder into frankfurters had little impact on their initial colour but did increase their yellowness during storage [169]. In a study on cooked beef taco, Wong et al. [170] reported a decrease in lightness (L*) with increasing level of mushroom (25–75%). In addition, the incorporation of 75% mushroom into the taco meat led to a lower redness (a*) than the all-meat control samples. These effects might arise due to the fact that the mushrooms were darker than the meat products, and because the myoglobin content in the final products was reduced after addition of the mushrooms. 

In contrast to the above studies, the addition of white jelly mushroom was found to slightly decrease the redness and increase the yellowness of cooked pork patties, which might be due to the transparent white colour of these mushrooms [126]. In general, the impact of mushrooms on muscle foods depends on the initial colour of the mushrooms and muscle foods, as well as any physical interactions or chemical reactions that can occur between them [171].

### 5.5. Mushrooms on the Microbiological Quality of Muscle Foods

Muscle foods contain high levels of macronutrients and micronutrients that spoilage or pathogenic microorganisms can utilize to grow. Therefore, it is important to have effective strategies to extend the shelf-life and ensure the safety of this type of food [172]. Many mushrooms and their constituents exhibit antibacterial and antifungal properties [154,173]. Consequently, their incorporation into muscle food products may have the added advantage of improving their safety and shelf-life. These antimicrobial properties have been attributed to a number of different mushroom constituents, including high-molecular weight (peptides and proteins) and low-molecular weight (terpenes, steroids, anthraquinones, benzoic acid derivatives and quinolones) compounds secreted by the mushroom fruiting body for its own survival [36,174]. Medicinal mushrooms like *Aleurodiscus*, *Coprinus*, *Clitocybe*, *Daedalea*, *Marasmius*, *Merulius*, *Pleurotus*, *Polyporus*, *Poria*, *Psathyrella*, and *Tricholoma* spp. have been used as a source of natural antibiotics for the treatment of various types of disease because of the immunomodulatory properties of β-glucans and the antibacterial properties of secondary metabolites [175,176]. In this section, we review studies on the antimicrobial properties of mushrooms and their constituents, with a focus on their application in muscle foods.

Chowdhury et al. [155] reported that extracts of *L. edodes*, *P. ostreatus* and *Hypsizigus tessulatus* demonstrated antimicrobial activity against all bacteria and fungi tested, with minimum inhibitory concentration (MIC) values ranging from 1 to 9 mg/mL. In this study, the *L. edodes* extracts were more effective than those from the other two mushroom species. Similarly, shiitake (*L. edodes*) mushroom extracts, isolated using organic solvents and supercritical fluids, exhibited antibacterial effects against pathogenic microorganisms such as *Streptococcus pyogenes* and *Staphylococcus aureus* [177], whereas extracts isolated using only supercritical fluid revealed antimicrobial activity against *Micrococcus luteus* and *Bacillus cereus* [178]. Extracts from *Pleurotus florida* mushroom have also been reported to exhibit strong inhibitory effects on the growth of both Gram-positive and Gram-negative bacteria and could, therefore, be considered as an alternative to traditional antibiotics [179].

Other studies suggest that mushrooms or their extracts can be used as natural preservatives that improve the shelf-life of foods by inhibiting the growth of spoilage microorganisms in muscle foods. Button mushrooms have been reported to exhibit antimicrobial activity in sutchi catfish patties, leading to a considerable extension in their shelf-life [71]. In another study, shitake (*L. edodes*) extracts were shown to exhibit good antimicrobial activity in fermented sausages, thereby extending their shelf-life and inhibiting the growth of pathogens such as *S. aureus*, *Listeria monocytogenes*, and *Escherichia coli* O157 [130]. Stojković et al. [180] reported that a methanolic extract from *Boletus aereus* was effective at controlling microbial growth against food-poisoning organisms in pork meat, including *S. aureus*, *L. monocytogenes*, *E. coli*, and *Salmonella Typhimurium.* Overall, these studies show that incorporating mushrooms into muscle foods, like meat or fish, may be an effective means of increasing their shelf-life and safety.

### 5.6. Mushroom on Sensory Attributes of Muscle Foods

The sensory attributes of food products play an important role in determining their quality and desirability. The overall sensory impression of muscle foods depends on their appearance, flavour, texture, and oral processing [181]. The incorporation of edible mushrooms into meat and fish products changes these physicochemical characteristics to an extent that depends on the type and level of mushrooms used, thereby altering their sensory attributes. Choe et al. [109] reported that incorporation of 1% winter mushroom powder into emulsion-type pork sausages led to better sensory scores (texture, flavour, and acceptability) than sausages with 2%. Wan Rosli et al. [134] reported that incorporation of 25% to 50% oyster mushrooms into chicken patties led to blended products that had similar sensory and liking scores as the all-meat control patties. Likewise, incorporation of 25% oyster mushrooms into meat patties was also shown to have no adverse effects on their sensory attributes [68]. 

Myrdal Miller et al. [122] found that incorporating 80% of white button mushrooms into reduced-salt ground beef tacos did not impact their flavour intensity. This study suggests that in some cases it is possible to include high levels of mushrooms into muscle food products while maintaining their desirable flavour profiles. This effect may be due to the relatively high content of free amino acids in mushrooms, which generate desirable meat-like umami, sweet, and bitter tastes [182,183]. The umami taste makes the edible mushrooms palatable and adaptable in most food preparations [44]. Chun et al. [125] reported that incorporating different levels (2%, 4% and 6%) of shiitake mushroom powder into pork patties increased their firmness, juiciness, flavour, and overall acceptability in a dose dependent manner. Similarly, incorporation of king oyster mushroom (30%, 40%, and 50%) into cuttlefish (*S*. *esculenta*) paste led to higher overall acceptability scores than the control paste [121]. A number of other studies have also reported no changes or an improvement in the sensory attributes of muscle food products after addition of mushrooms or their extracts, e.g., enoki mushroom powder (2%, 4% and 6%) to mutton nuggets [53] and ground white jelly mushrooms (10%, 20%, and 30% ground) to pork patties [126].

## 6. Conclusions

Mushrooms are a rich source of important nutrients and bioactive components, including proteins, fibres, vitamins, minerals, and nutraceuticals, while also being low in calories, sodium, fat, and cholesterol. They can also be produced more sustainably than meat products, with less damaging effects on the environment [6]. Moreover, they have many flavor and textural attributes that are compatible with meat and fish products. Consequently, they are an extremely valuable functional ingredient for creating muscle food products with enhanced nutritional and sustainability profiles. In particular, they can be used to create foods designed for a flexitarian diet where a fraction of the meat or fish in a product is reduced. 

There are many species of edible mushrooms that have not yet been explored for their potential application in food products. These novel mushroom species may have different nutritional, sensory, and environmental impacts than those exhibited by the species used so far. Consequently, there is a need for research on these other species. Moreover, further research is required to identify the mechanisms of action of different constituents in mushrooms, e.g., their antimicrobial activity, antioxidant activity, structures, textural attributes, and flavour profiles. Given the increasing demand of consumers for healthier and more sustainable foods, it is likely that the utilization of mushrooms within muscle foods is likely to continue to grow.

## Figures and Tables

**Table 2:** Effects of edible mushrooms and its parts on quality attribute of functional muscle food products.

Mushroom Variety and Level Used	Types of Muscle Food	Quality Parameters and Storage Conditions	Effects	Ref.
Dried mushroom (*Pleurotus ostreatus*) @ 4%, 8% or 12%	Beef patties	Quality attributes (stored at −18 to −20 °C for 6 months)	Increased protein, fat and ash contents, water holding capacity Decreased moisture, carbohydrate contents, pH value, tenderness, plasticity, cooking loss Better organoleptic properties of patties at 4 and 8% level	[67]
Mushroom (*Agaricus bisporus* or *P. ostreatus*) powder @ 5% or 10%	Traditional Turkish meatball	Sensory and physical (colour and texture) analysis	Imparted positive effect on hardness Meatball with *P. ostreatus* at 5% level was the best-liked	[108]
Mushroom (*Boletus edulis*) extract @ 1%, 3% or 5%	Beef burger	Antioxidant activities (stored at 4 °C for 8 days)	Protected lipid peroxidation Protected arachidonic (c20:4n6) and eicosapentaenoic (c20:5n3) acids Extended shelf-life	[119]
Winter mushroom (*Flammulina velutipes*) powder @ 0.5, 1%, 1.5% or 2%	Emulsion-type pork sausages	Quality parameters (pH, lipid oxidation, texture and sensory properties)	Increased water holding capacity and pH at >1% inclusion level Decreased exudation of fat and water from the sausages No adverse effect on colour and sensory properties at ≤1.5% inclusion level Could replace phosphates in meat products Had soft texture compared to control samples with phosphate	[109]
King oyster mushroom (*Pleurotus eryngii*) powder @10% or 15%	Chicken burger	Physical properties and sensory evaluation (stored at 6 ± 1 °C for 7 days)	Increased water holding capacity Lowered weight loss during cooking and thickness during storage at 15% inclusion level Improved juiciness and tenderness	[69]
Winter mushroom (*F. velutipes*) powder @ 0.5% or 1%	Low-salt chicken sausages	Sensory analysis (stored at 4 °C for 3 days)	Increased pH of the meat batter Inhibited lipid oxidation, softened texture Improved the nutritional quality No negative effect on colour and sensory properties	[70]
Blanched grey oyster mushroom (*P. sajor caju*) @ 25% or 50%	Chicken patties	Optical and textural properties (colour, textural and cooking characteristics)	Decreased lightness, yellowness but no change in redness values Had similar cooking yield and moisture contents compared to the all-meat control Less hardy, cohesive, chewy, and gummy	[120]
Mushroom (*F. velutipes*) extract @ 1 mL equivalent to 3 mg ergothioneine	Bigeye tuna (*Thunnus obesus*) meat	Antioxidative activity and anti-discolouration efficacy (frozen at −70 °C for 3 months)	Showed higher radical scavenging activity Suppressed lipid oxidation Significantly inhibited the formation of metmyoglobin Overcame the browning of big eye tuna meats up to 7 days of storage Plays an important role as a colour stabilizer of meats	[103]
King oyster mushroom @ 20%, 30%, 40%, or 50%	Cuttlefish (*Sepia esculenta*) surimi gel	Physico-chemical and sensory attributes	Significantly decreased the hardness, cohesiveness, and gumminess. Increased the springiness of paste Improved the nutritional properties and functionality of surimi gel	[121]
Mushroom (*A. bisporus*) powder @ 2%	Beef meat emulsion	Rheological and structural characteristics	Exhibited improvement in textural properties Improved viscoelastic behaviour Exhibited higher heat resistance and emulsion strength Provided a higher protein adsorption at the lipid interface, resulting in a well-ordered emulsion structure	[11]
Ground white mushroom (*A. bisporus*) @ 50% or 80%	Meat based dish (beef taco blend)	Nutritional quality and flavor profiles	Improved nutritional quality by reducing calories, saturated fat, and cholesterol Enhanced the flavor of the reduced salt version of the tacos Substitution with mushrooms @ 50% or even 80% can maintain flavour intensity scores even with less salt like that of all-meat, full salt control	[122]
Mushroom (*A. bisporus*) @ 0.5, 1% or 2%	Sucuk (dry-fermented product using beef meat (90%) and beef fat (10%)	Quality properties during ripening period at (18 ± 2 °C) up to 12 days (stored at 4 ± 1 °C in refrigerator for 60 days)	Prevented lipid oxidation Did not affect the sensory attributes Had a significant effect on the free fatty acids, peroxide value, appearance and colour indices Improve the product quality	[63]
*Pleurotus eryngii*, F. velutipes, *Lentinula edodes*, *Pleurotus cornucopiae* and processing waste of *F. velutipes* @ 1% of each extract separately	Yellow tail (*Seriola quinqueradiata*) dark muscle	Colour stabilizing effects (storage in cold room at 5 °C and replaced daily with fresh ice)	Had antioxidative activities against lipid oxidation and metmyoglobin formation. Exerted anti-discolouration efficacy and maintained bright-red colour after 2 days of ice storage	[123]
Oyster mushroom (*P. sajor caju*) powder @ 2%, 4% or 6%	Chicken sausages/frankfurters	Nutritional composition and textural properties (stored at −18 °C until analysis)	Enhanced dietary fibres up to 6.20% and ß-glucan up to 14.30% significantly Lowered fat content but unchanged adhesiveness and cohesiveness attributes	[124]
Button mushroom (*A. bisporus*) @ 15%	Patties from sutchi catfish (*Pangasius hypophthalmus*)	Physical, chemical, microbial and sensory properties (stored at 6 ± 2 °C for 20 days)	Increased the nutritional quality Lowered the lipid hydrolysis development rate Significantly lowered total volatile base nitrogen content Significantly reduced total plate count Improved shelf-life (up to 16 days) compared to control	[71]
Shiitake mushroom (*L. edodes*) powder @ 2%, 4% or 6%	Pork patties	Consumer acceptability and perception (stored at −20 °C up to one week)	Declined moisture loss Improved texture, juiciness, overall acceptability Increased mushroom flavour and juiciness while still deemed just as acceptable as the all-meat patties	[125]
White jelly mushroom (*Tremella fuciformis*) @ 10%, 20% or 30%	Pork patties	Quality and sensory characteristics	Better moisture and fat-holding capacity Had significantly higher cooking yield Had a higher lightness (64.31~67.23) and yellowness content than control No adverse effect on appearance, colour, flavour and texture	[126]
Mushroom (*A. bisporus*) @ 15% or 30%	Beef burger	Physico-chemical and sensory measurements (vacuum packed and stored at −18 °C)	Modified the texture, moisture and water activity Had a significantly lower hardness than the control Did not affect colour of the product	[127]
Mushroom (*F. velutipes*) extract @ 1% or 10%	Yellowtail (*Seriola quinqueradiata*)	Colour stability and lipid oxidation (stored at 0–2 °C for 4 days)	Remarkably suppressed the browning development in dark muscle Improved the quality of fish meat Significantly increased shelf-life of dark muscle in terms of colour Effectively controlled meat discolouration due to myoglobin and lipid oxidation	[103]
Button mushroom (*A. bisporus*) @ 5%, 10%, 15% or 20%	Fish paste	Texture profile analysis	Increased the elasticity, hardness, brittleness, and gumminess Decreased lightness and increased redness with increasing level Highest overall acceptance at 10% supplementation	[128]
King oyster mushroom (*P. eryngii*) @ 5%, 10%, 15% or 20%	Silver white croaker *(Pennahiaargentata* fried fish cake	Quality properties and sensory characteristics	Highest values in terms of strength, hardness, gumminess and brittleness Decreased the degree of lightness Increased redness and yellowness with increasing level Exhibited better colour, flavour and overall acceptance at 10% level	[129]
Dried mushroom (*A. bisporus*) @ 1%, 2% or 4%	Beef patties	Shelf-life and changes in lipid and protein oxidation (stored at 4 °C for 16 days)	Inhibited formation of lipid oxidation compounds Lowered malondialdehyde and volatile aldehydes compared to control samples Lowered the loss of thiols and tryptophan fluorescence @ 1% inclusion whereas at higher levels increased protein oxidation products compared to control Extended shelf-life in a concentration dependent manner	[116]
Enoki mushroom (*F. velutipes*) stem waste powder @ 2%, 4% or 6%	Goat meat nuggets	Physico-chemical qualities, antioxidant capacity and lipid oxidation stability (stored at 4 °C up to 12 days)	Improved cooking yield, mineral and dietary fibre content Extended shelf-life of meat products by inhibiting lipid oxidation No adverse effect on sensory attributes	[53]
Shiitake (*L. edodes*) by-products -stipes extract @ 0.3% or 0.6%	Fermented sausage (70% pork meat and 30% pork back-fat)	Quality characteristics, lipid oxidation and microbial stabilities (stored at 15 °C up to 30 days)	Lowered ultimate pH values with higher number of lactic acid bacteria Did not affect colour, texture and sensory quality Improved lipid oxidation and microbial stabilities as well as controlled the growth of pathogens (*Staphylococcus aureus, Listeria monocytogenes,* and *Escherichia coli* O157 at 0.6%)	[130]
Shiitake (*L. edodes*) by-products- stipes extract (aqueous or ethanolic)	Fermented sausages (80% pork ham and 20% pork fat)	Antioxidant and microbial abilities (stored at 15 °C up to 40 days)	Had higher antioxidant activities and inhibitory capacity against lipid oxidation Had stronger antimicrobial activities against pathogens Ethanolic extract partly showed beneficial effects on the flavour and taste improvements than control Had significantly higher colour, flavour, taste and acceptability scores compared to control	[107]
Immature white button mushroom (*A. bisporus*) @ 10%, 20%, 30%, 40% or 50%	Ground beef (80/20 blend)	Physical and sensory characteristics (stored at −18 °C in between sheets of wax paper in plastic bags)	No difference in yield, liking scores, lightness (L* value), and red colour (a* value) Increased the moisture and yellow colour (b* value) Decreased the mechanical properties, sodium content, and fat content of the final products Potential to reduce sodium contents in meat products providing a healthier product	[131]

## Data Availability

Not applicable.

## References

[1] Lawrie R.A., Ledward D. (2006). Lawrie’s Meat Science.

[2] Corpet D.E. (2011). Red meat and colon cancer: Should we become vegetarians, or can we make meat safer?. Meat Sci..

[3] Young J.F., Therkildsen M., Ekstrand B., Che B.N., Larsen M.K., Oksbjerg N., Stagsted J. (2013). Novel aspects of health promoting compounds in meat. Meat Sci..

[4] McClements D.J., Barrangou R., Hill C., Kokini J.L., Ann Lila M., Meyer A.S., Yu L. (2021). Building a Resilient, Sustainable, and Healthier Food Supply through Innovation and Technology. Annu. Rev. Food Sci. Technol..

[5] WHO (2004). Global Strategy on Diet, Physical Activity and Health.

[6] Poore J., Nemecek T. (2018). Reducing food’s environmental impacts through producers and consumers. Science.

[7] McClements D.J., Das A.K., Dhar P., Nanda P.K., Chatterjee N. (2021). Nanoemulsion-based technologies for delivering natural plant-based antimicrobials in foods. Front. Sustain. Food Syst..

[8] Das A.K., Nanda P.K., Madane P., Biswas S., Das A., Zhang W., Lorenzo J.M. (2020). A comprehensive review on antioxidant dietary fibre enriched meat-based functional foods. Trends Food Sci. Technol..

[9] Bano Z., Rajarathnam S. (1988). Pleurotus mushrooms. Part II. Chemical composition, nutritional value, post-harvest physiology, preservation, and role as human food. Crit. Rev. Food Sci. Nutr..

[10] Hernández-Martínez R., Navarro-Blasco I. (2015). Surveillance of aflatoxin content in dairy cow feedstuff from Navarra (Spain). Anim. Feed Sci. Technol..

[11] Kurt A., Gençcelep H. (2018). Enrichment of meat emulsion with mushroom (*Agaricus bisporus*) powder: Impact on rheological and structural characteristics. J. Food Eng..

[12] Lu X., Brennan M.A., Narciso J., Guan W., Zhang J., Yuan L., Serventi L., Brennan C.S. (2020). Correlations between the phenolic and fibre composition of mushrooms and the glycaemic and textural characteristics of mushroom enriched extruded products. LWT.

[13] Lakhanpal T.N., Rana M. (2005). Medicinal and nutraceutical genetic resources of mushrooms. Plant Genet. Resour..

[14] Chang S.T. (2009). Overview of Mushroom Cultivation and Utilization as Functional Foods. Mushrooms as Functional Foods.

[15] Wang H.X., Liu W.K., Ng T.B., Ooi V.E.C., Chang S.T. (1996). The immunomodulatory and antitumor activities of lectins from the mushroom Tricholoma mongolicum. Immunopharmacology.

[16] Liu F., Ooi V.E.C., Liu W.K., Chang S.T. (1996). Immunomodulation and antitumor activity of polysaccharide-protein complex from the culture filtrates of a local edible mushroom, *Tricholoma lobayense*. Gen. Pharmacol..

[17] Tam S.C., Yip K.P., Fung K.P., Chang S.T. (1986). Hypotensive and renal effects of an extract of the edible mushroom *Pleurotus sajor-caju*. Life Sci..

[18] Jana P., Acharya K. (2020). Mushroom: A New Resource for Anti-Angiogenic Therapeutics. Food Rev. Int..

[19] Spencer M., Guinard J.X. (2018). The Flexitarian Flip^TM^: Testing the modalities of flavor as sensory strategies to accomplish the shift from meat-centered to vegetable-forward mixed dishes. J. Food Sci..

[20] Lang M. (2020). Consumer acceptance of blending plant-based ingredients into traditional meat-based foods: Evidence from the meat-mushroom blend. Food Qual. Prefer..

[21] Guinard J.X., Myrdal Miller A., Mills K., Wong T., Lee S.M., Sirimuangmoon C., Schaefer S.E., Drescher G. (2016). Consumer acceptance of dishes in which beef has been partially substituted with mushrooms and sodium has been reduced. Appetite.

[22] Summers A., Ezike A., Smith P., Frutchey R., Leslie L., Paredes S., Alvarado C., Karani S., Taylor J., Cheskin L. (2017). Acceptance of a mushroom-soy-beef blended burger among school-aged children. Heal. Behav. Policy Rev..

[23] Kumar P., Chatli M.K., Mehta N., Singh P., Malav O.P., Verma A.K. (2017). Meat analogues: Health promising sustainable meat substitutes. Crit. Rev. Food Sci. Nutr..

[24] He J., Evans N.M., Liu H., Shao S. (2020). A review of research on plant-based meat alternatives: Driving forces, history, manufacturing, and consumer attitudes. Compr. Rev. Food Sci. Food Saf..

[25] Raghavendra V.B., Venkitasamy C., Pan Z., Nayak C. (2017). Functional foods from mushroom. Microbial Functional Foods and Nutraceuticals.

[26] Chang S.T. (2006). The world mushroom industry: Trends and technological development. Int. J. Med. Mushrooms.

[27] Feeney M.J., Dwyer J., Hasler-Lewis C.M., Milner J.A., Noakes M., Rowe S., Wach M., Beelman R.B., Caldwell J., Cantorna M.T. (2014). Mushrooms and health summit proceedings. J. Nutr..

[28] Royse D.J., Baars J., Tan Q., Zied D.C., Pardo-Giminez A. (2017). Current overview of mushroom production in the world. Edible and Medicinal Mushrooms: Technology and Applications.

[29] Beulah G.H., Margret A.A., Nelson J. (2013). Marvelous Medicinal Mushrooms. Int. J. Pharm. Biol. Sci..

[30] Kumar K. (2018). Nutraceutical Potential and Processing Aspects of Oyster Mushrooms (*Pleurotus* Species). Curr. Nutr. Food Sci..

[31] Papoutsis K., Grasso S., Menon A., Brunton N.P., Lyng J.G., Jacquier J.C., Bhuyan D.J. (2020). Recovery of ergosterol and vitamin D2 from mushroom waste—Potential valorization by food and pharmaceutical industries. Trends Food Sci. Technol..

[32] Zhang R., Li X., Fadel J.G. (2002). Oyster mushroom cultivation with rice and wheat straw. Bioresour. Technol..

[33] Wu S.R., Zhao C.Y., Hou B., Tai L.M., Gui M.Y. (2013). Analysis on Chinese edible fungus production area layout of nearly five years. Edible Fungi China.

[34] Fasseas M.K.K., Mountzouris K.C.C., Tarantilis P.A.A., Polissiou M., Zervas G. (2008). Antioxidant activity in meat treated with oregano and sage essential oils. Food Chem..

[35] El Sohaimy S. (2012). Functional foods and nutraceuticals-modern approach to food science. World Appl. Sci. J..

[36] Reis F.S., Martins A., Vasconcelos M.H., Morales P., Ferreira I.C.F.R. (2017). Functional foods based on extracts or compounds derived from mushrooms. Trends Food Sci. Technol..

[37] Hasler C.M. (2002). Functional Foods: Benefits, Concerns and Challenges—A Position Paper from the American Council on Science and Health. J. Nutr..

[38] Asgar M.A., Fazilah A., Huda N., Bhat R., Karim A.A. (2010). Nonmeat protein alternatives as meat extenders and meat analogs. Compr. Rev. Food Sci. Food Saf..

[39] Chadha K., Sharma S., Chadha K.L. (1995). Mushroom research in India-History, Infrastructure and Achievements. Advances in Horticulture.

[40] Kakon A., Choudhury M.B.K., Saha S. (2012). Mushroom is an ideal food supplement. J. Dhaka Natl. Med. Coll. Hosp..

[41] Oyetayo F.L., Akindahunsi A.A., Oyetayo V.O. (2007). Chemical profile and amino acids composition of edible mushrooms *Pleurotus sajor-caju*. Nutr. Health.

[42] Prasad S., Rathore H., Sharma S., Yadav A.S. (2015). Medicinal Mushrooms as a Source of Novel Functional Food. Int. J. Food Sci. Nutr. Diet..

[43] Rathore H., Prasad S., Sharma S. (2017). Mushroom nutraceuticals for improved nutrition and better human health: A review. Pharma Nutr..

[44] Zhang Y., Venkitasamy C., Pan Z., Wang W. (2013). Recent developments on umami ingredients of edible mushrooms—A review. Trends Food Sci. Technol..

[45] Jeng-Leun M. (2005). The Umami Taste of Edible and Medicinal Mushrooms. Int. J. Med. Mushrooms.

[46] Dunkel A., Köster J., Hofmann T. (2007). Molecular and sensory characterization of γ-glutamyl peptides as key contributors to the kokumi taste of edible beans (*Phaseolus vulgaris* L.). J. Agric. Food Chem..

[47] Kong Y., Yang X., Ding Q., Zhang Y.Y., Sun B.G., Chen H.T., Sun Y. (2017). Comparison of non-volatile umami components in chicken soup and chicken enzymatic hydrolysate. Food Res. Int..

[48] Kong Y., Zhang L.L., Zhao J., Zhang Y.Y., Sun B.G., Chen H.T. (2019). Isolation and identification of the umami peptides from shiitake mushroom by consecutive chromatography and LC-Q-TOF-MS. Food Res. Int..

[49] Feng T., Wu Y., Zhang Z., Song S., Zhuang H., Xu Z., Yao L., Sun M. (2019). Purification, identification, and sensory evaluation of kokumi peptides from agaricus bisporus mushroom. Foods.

[50] Ueda Y., Yonemitsu M., Tsubuku T., Sakaguchi M., Miyajima R. (1997). Flavor characteristics of glutathione in raw and cooked foodstuffs. Biosci. Biotechnol. Biochem..

[51] Chang S.T. (2003). Buswell Mushroom Production. Biotechnology, Vol. VII. Encyclopedia of Life Support Systems.

[52] Synytsya A., Míčková K., Jablonský I., Sluková M., Čopíková J. (2008). Mushrooms of Genus Pleurotus as a source of dietary fibres and glucans for food supplements. Czech J. Food Sci..

[53] Banerjee D.K., Das A.K., Banerjee R., Pateiro M., Nanda P.K., Gadekar Y.P., Biswas S., McClements D.J., Lorenzo J.M. (2020). Application of enoki mushroom (*Flammulina velutipes*) stem wastes as functional ingredients in processed meat. Foods.

[54] Due E.A., Michel K.D., Digbeu Y.D. (2016). Physicochemical and Functional Properties of Flour from the Wild Edible Mushroom Termitomyces heimii Natarajan Harvested in Côte d’Ivoire. Turkish J. Agric. Food Sci. Technol..

[55] Chaturvedi V.K., Agarwal S., Gupta K.K., Ramteke P.W., Singh M.P. (2018). Medicinal mushroom: Boon for therapeutic applications. 3 Biotech.

[56] Sawangwan T., Wansanit W., Pattani L., Noysang C. (2018). Study of prebiotic properties from edible mushroom extraction. Agric. Nat. Resour..

[57] Aida F.M.N.A., Shuhaimi M., Yazid M., Maaruf A.G. (2009). Mushroom as a potential source of prebiotics: A review. Trends Food Sci. Technol..

[58] Kozarski M., Klaus A., Jakovljevic D., Todorovic N., Vunduk J., Petrović P., Niksic M., Vrvic M.M., Van Griensven L. (2015). Antioxidants of edible mushrooms. Molecules.

[59] Randive S.D. (2012). Cultivation and study of growth of oyster mushroom on different agricultural waste substrate and its nutrient analysis. Adv. Appl. Sci. Res..

[60] Valverde M.E., Hernández-Pérez T., Paredes-López O. (2015). Edible mushrooms: Improving human health and promoting quality life. Int. J. Microbiol..

[61] Friedman M. (2016). Mushroom Polysaccharides: Chemistry and Antiobesity, Antidiabetes, Anticancer, and Antibiotic Properties in Cells, Rodents, and Humans. Foods.

[62] Cardwell G., Bornman J.F., James A.P., Black L.J. (2018). A review of mushrooms as a potential source of dietary vitamin D. Nutrients.

[63] Gençcelep H. (2012). The effect of using dried mushroom (*Agaricus bisporus*) on lipid oxidation and color properties of sucuk. J. Food Biochem..

[64] Ferreira I., Barros L., Abreu R. (2009). Antioxidants in wild mushrooms. Curr. Med. Chem..

[65] Ho L.-H., Asyikeen Zulkifli N., Tan T.-C. (2020). Edible Mushroom: Nutritional Properties, Potential Nutraceutical Values, and Its Utilisation in Food Product Development. An Introduction to Mushroom.

[66] Marçal S., Sousa A.S., Taofiq O., Antunes F., Morais A.M.M.B., Freitas A.C., Barros L., Ferreira I.C.F.R., Pintado M. (2021). Impact of postharvest preservation methods on nutritional value and bioactive properties of mushrooms. Trends Food Sci. Technol..

[67] El-Refai A., El-Zeiny A.R., Rabo E.A. (2014). Quality attributes of mushroom-beef patties as a functional meat product. J. Hyg. Eng. Des..

[68] Wan Rosli W.I., Solihah M.A. (2014). Nutritional composition and sensory properties of oyster mushroom-based patties packed with biodegradable packaging. Sains Malays..

[69] Dosh K.S., Tawfiq N.N., Jabbar S.H. (2016). Preparation of modified chicken burger by partial replacement of chicken meat with powdered of oyster mushroom and study its physical and sensory Properties. Iraqi J. Agric. Sci..

[70] Jo K., Lee J., Jung S. (2018). Quality characteristics of low-salt chicken sausage supplemented with a winter mushroom powder. Korean J. Food Sci. Anim. Resour..

[71] Nayak P.C., Raju C.V., Lakshmisha I.P., Singh R.R., Sofi F.R. (2015). Influence of Button mushroom (*Agaricus bisporus*) on quality and refrigerated storage stability of patties prepared from sutchi catfish (*Pangasius hypophthalmus*). J. Food Sci. Technol..

[72] Mau J.L., Lin H.C., Ma J.T., Song S.F. (2001). Non-volatile taste components of several speciality mushrooms. Food Chem..

[73] Heleno S.A., Barros L., Sousa M.J., Martins A., Santos-Buelga C., Ferreira I.C.F.R. (2011). Targeted metabolites analysis in wild *Boletus* species. LWT Food Sci. Technol..

[74] Michael H.W., Bultosa G., Pant L.M. (2011). Nutritional contents of three edible oyster mushrooms grown on two substrates at Haramaya, Ethiopia, and sensory properties of boiled mushroom and mushroom sauce. Int. J. Food Sci. Technol..

[75] Vaz J.A., Barros L., Martins A., Santos-Buelga C., Vasconcelos M.H., Ferreira I.C.F.R. (2011). Chemical composition of wild edible mushrooms and antioxidant properties of their water soluble polysaccharidic and ethanolic fractions. Food Chem..

[76] Teklit G.A. (2015). Chemical composition and nutritional value of the most widely used mushrooms cultivated in Mekelle Tigray Ethiopia. J. Nutr. Food Sci..

[77] Heleno S.A., Barros L., Sousa M.J., Martins A., Ferreira I.C.F.R. (2009). Study and characterization of selected nutrients in wild mushrooms from Portugal by gas chromatography and high performance liquid chromatography. Microchem. J..

[78] Crisan E.V., Sands A. (1978). Nutritional value. Biol. Cultiv. Edible Mushrooms.

[79] Pereira M.C., Steffens R.S., Jablonski A., Hertz P.F., de Rios A.O., Vizzotto M., Flôres S.H. (2012). Characterization and Antioxidant Potential of Brazilian Fruits from the Myrtaceae Family. J. Agric. Food Chem..

[80] Ghosh K. (2016). A review: Edible mushrooms as source of dietary fiber and its healtheffects. J. Phys. Sci..

[81] Chang S.T., Buswell J.A. (1996). Mushroom nutriceuticals. World J. Microbiol. Biotechnol..

[82] Ma G., Yang W., Zhao L., Pei F., Fang D., Hu Q. (2018). A critical review on the health promoting effects of mushrooms nutraceuticals. Food Sci. Hum. Wellness.

[83] El Enshasy H.A., Hatti-Kaul R. (2013). Mushroom immunomodulators: Unique molecules with unlimited applications. Trends Biotechnol..

[84] Patel S., Goyal A. (2012). Recent developments in mushrooms as anti-cancer therapeutics: A review. 3 Biotech.

[85] Ina K., Kataoka T., Ando T. (2013). The Use of Lentinan for Treating Gastric Cancer. Anticancer Agents Med. Chem..

[86] Nowacka-Jechalke N., Olech M., Nowak R. (2018). Mushroom polyphenols as chemopreventive agents. Polyphenols: Prevention and Treatment of Human Disease.

[87] Ruthes A.C., Smiderle F.R., Iacomini M. (2015). D-Glucans from edible mushrooms: A review on the extraction, purification and chemical characterization approaches. Carbohydr. Polym..

[88] Borchers A.T., Krishnamurthy A., Keen C.L., Meyers F.J., Gershwin M.E. (2008). The immunobiology of mushrooms. Exp. Biol. Med..

[89] Zhang C.-X., Ho S.C., Chen Y.-M., Lin F.-Y., Fu J.-H., Cheng S.-Z. (2009). Meat and egg consumption and risk of breast cancer among Chinese women. Cancer Causes Control.

[90] Khan I., Huang G., Li X., Leong W., Xia W., Hsiao W.L.W. (2018). Mushroom polysaccharides from *Ganoderma lucidum* and *Poria cocos* reveal prebiotic functions. J. Funct. Foods.

[91] Jayachandran M., Xiao J., Xu B. (2017). A critical review on health promoting benefits of edible mushrooms through gut microbiota. Int. J. Mol. Sci..

[92] Yin C., Noratto G.D., Fan X., Chen Z., Yao F., Shi D., Gao H. (2020). The Impact of Mushroom Polysaccharides on Gut Microbiota and Its Beneficial Effects to Host: A Review. Carbohydr. Polym..

[93] Cheung M.K., Yue G.G.L., Chiu P.W.Y., Lau C.B.S. (2020). A Review of the Effects of Natural Compounds, Medicinal Plants, and Mushrooms on the Gut Microbiota in Colitis and Cancer. Front. Pharmacol..

[94] Chou W.T., Sheih I.C., Fang T.J. (2013). The applications of polysaccharides from various mushroom wastes as prebiotics in different systems. J. Food Sci..

[95] Synytsya A., Mickova K., Jablonsky I., Spevacek J., Erban V., Kovarikova E., Copikova J. (2009). Glucans from fruit bodies of cultivated mushrooms *Pleurotus ostreatus* and *Pleurotus eryngii*: Structure and potential prebiotic activity. Carbohydr. Polym..

[96] Arora T., Sharma R. (2011). Fermentation potential of the gut microbiome: Implications for energy homeostasis and weight management. Nutr. Rev..

[97] Chang C.J., Lin C.S., Lu C.C., Martel J., Ko Y.F., Ojcius D.M., Tseng S.F., Wu T.R., Chen Y.Y.M., Young J.D. (2015). Ganoderma lucidum reduces obesity in mice by modulating the composition of the gut microbiota. Nat. Commun..

[98] Delzenne N.M., Bindels L.B. (2015). Gut microbiota: *Ganoderma lucidum*, a new prebiotic agent to treat obesity?. Nat. Rev. Gastroenterol. Hepatol..

[99] Lo H.C., Wasser S.P. (2011). Medicinal mushrooms for glycemic control in diabetes mellitus: History, current status, future perspectives, and unsolved problems (review). Int. J. Med. Mushrooms.

[100] Ouzouni P.K., Petridis D., Koller W.D., Riganakos K.A. (2009). Nutritional value and metal content of wild edible mushrooms collected from West Macedonia and Epirus, Greece. Food Chem..

[101] Lee Y.L., Jian S.Y., Mau J.L. (2009). Composition and non-volatile taste components of *Hypsizigus marmoreus*. LWT Food Sci. Technol..

[102] Ulziijargal E., Yang J.H., Lin L.Y., Chen C.P., Mau J.L. (2013). Quality of bread supplemented with mushroom mycelia. Food Chem..

[103] Bao H.N.D., Ushio H., Ohshima T. (2009). Antioxidative activities of mushroom (*Flammulina velutipes*) extract added to bigeye tuna meat: Dose-dependent efficacy and comparison with other biological antioxidants. J. Food Sci..

[104] Biao Y., Chen X., Wang S., Chen G., Mcclements D.J., Zhao L. (2020). Impact of mushroom (*Pleurotus eryngii*) flour upon quality attributes of wheat dough and functional cookies-baked products. Food Sci. Nutr..

[105] Tuley L. (1996). Swell time for dehydrated vegetables. Int. Food Ingred..

[106] Gothandapani L., Parvathi K., John Kennedy Z. (1997). Evaluation of different methods of drying on the quality of oyster mushroom (*Pleurotus* sp.). Dry. Technol..

[107] Van Ba H., Seo H.W., Cho S.H., Kim Y.S., Kim J.H., Ham J.S., Park B.Y., Pil-Nam S. (2017). Effects of extraction methods of shiitake by-products on their antioxidant and antimicrobial activities in fermented sausages during storage. Food Control.

[108] Süfer Ö., Bozok F., Demir H. (2016). Usage of Edible Mushrooms in Various Food Products. Turkish J. Agric. Food Sci. Technol..

[109] Choe J., Lee J., Jo K., Jo C., Song M., Jung S. (2018). Application of winter mushroom powder as an alternative to phosphates in emulsion-type sausages. Meat Sci..

[110] Gençcelep H., Uzun Y., Tunçtürk Y., Demirel K. (2009). Determination of mineral contents of wild-grown edible mushrooms. Food Chem..

[111] Vetter J. (2003). Data on sodium content of common edible mushrooms. Food Chem..

[112] Seeger R., Trumpfheller S., Schweinshaut P. (1983). On the occurrence of sodium in fungi. Dtsch. Leb..

[113] Inguglia E.S., Zhang Z., Tiwari B.K., Kerry J.P., Burgess C.M. (2017). Salt reduction strategies in processed meat products—A review. Trends Food Sci. Technol..

[114] Chrysant S.G. (2016). Effects of high salt intake on blood pressure and cardiovascular disease: The role of COX inhibitors. Clin. Cardiol..

[115] Ruusunen M., Puolanne E. (2005). Reducing sodium intake from meat products. Meat Sci..

[116] Alnoumani H., Ataman Z.A., Were L. (2017). Lipid and protein antioxidant capacity of dried *Agaricus bisporus* in salted cooked ground beef. Meat Sci..

[117] Bao H.N.D., Shinomiya Y., Ikeda H., Ohshima T. (2009). Preventing discoloration and lipid oxidation in dark muscle of yellowtail by feeding an extract prepared from mushroom (*Flammulina velutipes*) cultured medium. Aquaculture.

[118] Encarnacion A.B., Fagutao F., Hirono I., Ushio H., Ohshima T. (2010). Effects of ergothioneine from mushrooms (*Flammulina velutipes*) on melanosis and lipid oxidation of kuruma shrimp (*Marsupenaeus japonicus*). J. Agric. Food Chem..

[119] Barros L., Barreira J.C.M., Grangeia C., Batista C., Cadavez V.A.P., Ferreira I.C.F.R. (2011). Beef burger patties incorporated with Boletus edulis extracts: Lipid peroxidation inhibition effects. Eur. J. Lipid Sci. Technol..

[120] Wan Rosli W.I., Solihah M.A., Mohsin S.S.J. (2011). On the ability of oyster mushroom (*Pleurotus sajor-caju*) confering changes in proximate composition and sensory evaluation of chicken patty. Int. Food Res. J..

[121] Chung S.I., Kim S.Y., Nam Y.J., Kang M.Y. (2010). Development of surimi gel from king oyster mushroom and cuttlefish meat paste. Food Sci. Biotechnol..

[122] Myrdal Miller A., Mills K., Wong T., Drescher G., Lee S.M., Sirimuangmoon C., Schaefer S., Langstaff S., Minor B., Guinard J.X. (2014). Flavor-enhancing properties of mushrooms in meat-based dishes in which sodium has been reduced and meat has been partially substituted with mushrooms. J. Food Sci..

[123] Bao H.N.D., Osako K., Ohshima T. (2010). Value-added use of mushroom ergothioneine as a colour stabilizer in processed fish meats. J. Sci. Food Agric..

[124] Wan Rosli W.I., Nor Maihiza M.S., Raushan M. (2015). The ability of oyster mushroom in improving nutritional composition, β-glucan and textural properties of chicken frankfurter. Int. Food Res. J..

[125] Chun S., Chambers E., Chambers D. (2005). Perception of pork patties with shiitake (*Lentinus edode* P.) mushroom powder and sodium tripolyphosphate as measured by Korean and United States consumers. J. Sens. Stud..

[126] Cha M.H., Heo J.Y., Lee C., Lo Y.M., Moon B. (2014). Quality and sensory characterization of white jelly mushroom (*Tremella fuciformis*) as a meat substitute in pork patty formulation. J. Food Process. Preserv..

[127] Patinho I., Saldaña E., Selani M.M., de Camargo A.C., Merlo T.C., Menegali B.S., de Souza Silva A.P., Contreras-Castillo C.J. (2019). Use of *Agaricus bisporus* mushroom in beef burgers: Antioxidant, flavor enhancer and fat replacing potential. Food Prod. Process. Nutr..

[128] Ha J.-U., Koo S.-G., Lee H.-Y., Hwang Y.-M., Lee S.-C. (2001). Physical properties of fish paste containing *Agaricus bisporus*. Korean J. Food Sci. Technol..

[129] Kim S.Y., Son M.H., Ha J.U., Lee S.C. (2003). Preparation and characterization of friend surimi gel containing king oyster mushroom (*Pleurotus eryngii*). J. Korean Soc. Food Sci. Nutr..

[130] Van Ba H., Seo H.W., Cho S.H., Kim Y.S., Kim J.H., Ham J.S., Park B.Y., Pil Nam S. (2016). Antioxidant and anti-foodborne bacteria activities of shiitake by-product extract in fermented sausages. Food Control.

[131] Wong K.M., Corradini M.G., Autio W., Kinchla A.J. (2019). Sodium reduction strategies through use of meat extenders (white button mushrooms vs. textured soy) in beef patties. Food Sci. Nutr..

[132] Biswas S., Banerjee R., Bhattacharyya D., Patra G., Das A.K., Das S.K. (2019). Technological investigation into duck meat and its products—A potential alternative to chicken. Worlds. Poult. Sci. J..

[133] Lorenzo J.M., Pateiro M. (2013). Influence of type of muscles on nutritional value of foal meat. Meat Sci..

[134] Wan Rosli W.I., Solihah M.A., Aishah M., Nik Fakurudin N.A., Mohsin S.S.J. (2011). Colour, textural properties, cooking characteristics and fibre content of chicken patty added with oyster mushroom (*Pleurotus sajor-caju*). Int. Food Res. J..

[135] Bao H.N.D., Ushio H., Ohshima T. (2008). Antioxidative activity and antidiscoloration efficacy of ergothioneine in mushroom (*Flammulina velutipes*) extract added to beef and fish meats. J. Agric. Food Chem..

[136] Ito H., Ueno H., Kikuzaki H. (2017). Construction of a free-form amino acid database for vegetables and mushrooms. Integr. Food, Nutr. Metab..

[137] Ko M.S., Kim S.A. (2007). Sensory and physicochemical characteristics of jeungpyun with *Pleurotus eryngii* powder. Korean J. Food Sci. Technol..

[138] Guyon C., Meynier A., de Lamballerie M. (2016). Protein and lipid oxidation in meat: A review with emphasis on high-pressure treatments. Trends Food Sci. Technol..

[139] Das A.K., Nanda P.K., Chowdhury N.R., Dandapat P., Gagaoua M., Chauhan P., Pateiro M., Lorenzo J.M. (2021). Application of Pomegranate by-Products in Muscle Foods: Oxidative Indices, Colour Stability, Shelf Life and Health Benefits. Molecules.

[140] Domínguez R., Pateiro M., Gagaoua M., Barba F.J., Zhang W., Lorenzo J.M. (2019). A comprehensive review on lipid oxidation in meat and meat products. Antioxidants.

[141] Lund M.N., Heinonen M., Baron C.P., Estevez M. (2011). Protein oxidation in muscle foods: A review. Mol. Nutr. Food Res..

[142] Miller D.K., Gomez-Basauri J.V., Smith V.L., Kanner J., Miller D.D. (1994). Dietary Iron in Swine Rations Affects Nonheme Iron and TBARS in Pork Skeletal Muscles. J. Food Sci..

[143] Soladoye O.P., Juárez M.L., Aalhus J.L., Shand P., Estévez M. (2015). Protein oxidation in processed meat: Mechanisms and potential implications on human health. Compr. Rev. Food Sci. Food Saf..

[144] Lobo F., Ventanas S., Morcuende D., Estévez M. (2016). Underlying chemical mechanisms of the contradictory effects of NaCl reduction on the redox-state of meat proteins in fermented sausages. LWT-Food Sci. Technol..

[145] Ferioli F., Dutta P.C., Caboni M.F. (2010). Cholesterol and lipid oxidation in raw and pan-fried minced beef stored under aerobic packaging. J. Sci. Food Agric..

[146] Gatellier P., Kondjoyan A., Portanguen S., Santé-Lhoutellier V. (2010). Effect of cooking on protein oxidation in n-3 polyunsaturated fatty acids enriched beef. Implication on nutritional quality. Meat Sci..

[147] Maqsood S., Benjakul S., Abushelaibi A., Alam A. (2014). Phenolic compounds andplant phenolic extracts as natural antioxidants in prevention of lipid oxidation in seafood: A detailed review. Compr. Rev. Food Sci. Food Saf..

[148] Falowo A.B., Fayemi P.O., Muchenje V. (2014). Natural antioxidants against lipid–protein oxidative deterioration in meat and meat products: A review. Food Res. Int..

[149] Das A.K., Rajkumar V., Nanda P.K., Chauhan P., Pradhan S.R., Biswas S. (2016). Antioxidant efficacy of litchi (*Litchi chinensis* Sonn.) pericarp extract in sheep meat nuggets. Antioxidants.

[150] Munekata P.E.S., Rocchetti G., Pateiro M., Lucini L., Domínguez R., Lorenzo J.M. (2020). Addition of plant extracts to meat and meat products to extend shelf-life and health-promoting attributes: An overview. Curr. Opin. Food Sci..

[151] Das A.K., Rajkumar V., Verma A.K. (2015). Bael pulp residue as a new source of antioxidant dietary fiber in goat meat nuggets. J. Food Process. Preserv..

[152] Lorenzo J.M., Pateiro M., Domínguez R., Barba F.J., Putnik P., Kovačević D.B., Shpigelman A., Granato D., Franco D. (2018). Berries extracts as natural antioxidants in meat products: A review. Food Res. Int..

[153] Ren X., Wang J., Huang L., Cheng K., Zhang M., Yang H. (2020). Comparative studies on bioactive compounds, ganoderic acid biosynthesis, and antioxidant activity of pileus and stipes of lingzhi or reishi medicinal mushroom, ganoderma lucidum (*Agaricomycetes*) fruiting body at different growth stages. Int. J. Med. Mushrooms.

[154] Shen H.S., Shao S., Chen J.C., Zhou T. (2017). Antimicrobials from Mushrooms for Assuring Food Safety. Compr. Rev. Food Sci. Food Saf..

[155] Chowdhury H.M.H., Kubra K., Ahmed R.R. (2015). Screening of antimicrobial, antioxidant properties and bioactive compounds of some edible mushrooms cultivated in Bangladesh. Ann. Clin. Microbiol. Antimicrob..

[156] Lee D.G., Lee J., Jo K., Lee C.W., Lee H.J., Jo C., Jung S. (2017). Improved oxidative stability of enhanced pork loins using red perilla extract. Korean J. Food Sci. Anim. Resour..

[157] Yen M.T., Tseng Y.H., Li R.C., Mau J.L. (2007). Antioxidant properties of fungal chitosan from shiitake stipes. LWT Food Sci. Technol..

[158] Zhang N., Chen H., Zhang Y., Ma L., Xu X. (2013). Comparative studies on chemical parameters and antioxidant properties of stipes and caps of shiitake mushroom as affected by different drying methods. J. Sci. Food Agric..

[159] Fu H.Y., Shieh D.E., Ho C.T. (2002). Antioxidant and free radical scavenging activities of edible mushrooms. J. Food Lipids.

[160] Elmastas M., Isildak O., Turkekul I., Temur N. (2007). Determination of antioxidant activity and antioxidant compounds in wild edible mushrooms. J. Food Compos. Anal..

[161] Kosanic M., Rankovic B., Dasic M. (2013). Antioxidant and antimicrobial properties of mushrooms. Bulg. J. Agric. Sci..

[162] Hameed A., Hussain S.A., Ijaz M.U., Ullah S., Muhammad Z., Suleria H.A.R., Song Y. (2020). Antioxidant activity of polyphenolic extracts of filamentous fungus Mucor circinelloides (WJ11): Extraction, characterization and storage stability of food emulsions. Food Biosci..

[163] Coggins P.C., Nollet L. (2007). Attributes of muscle foods: Color, texture, flavor. Handbook of Meat, Poultry and Seafood Quality.

[164] Han M., Bertram H.C. (2017). Designing healthier comminuted meat products: Effect of dietary fibers on water distribution and texture of a fat-reduced meat model system. Meat Sci..

[165] Madane P., Das A.K., Nanda P.K., Bandyopadhyay S., Jagtap P., Shewalkar A., Maity B. (2020). Dragon fruit (*Hylocereus undatus*) peel as antioxidant dietary fibre on quality and lipid oxidation of chicken nuggets. J. Food Sci. Technol..

[166] Chauhan P., Das A.K., Das A., Bhattacharya D., Nanda P.K. (2018). Effect of black cumin and arjuna fruit extract on lipid oxidation in pork nuggets during refrigerated storage. J. Meat Sci..

[167] Madane P., Das A.K., Pateiro M., Nanda P.K., Bandyopadhyay S., Jagtap P., Barba F.J., Shewalkar A., Maity B., Lorenzo J.M. (2019). Drumstick (*Moringa oleifera*) flower as an antioxidant dietary fibre in chicken meat nuggets. Foods.

[168] Baron C.P., Andersen H.J. (2002). Myoglobin-induced lipid oxidation. A review. J. Agric. Food Chem..

[169] Pil-Nam S., Park K.M., Kang G.H., Cho S.H., Park B.Y., Van-Ba H. (2015). The impact of addition of shiitake on quality characteristics of frankfurter during refrigerated storage. LWT Food Sci. Technol..

[170] Wong K.M., Decker E.A., Autio W.R., Toong K., DiStefano G., Kinchla A.J. (2017). Utilizing Mushrooms to Reduce Overall Sodium in Taco Filling Using Physical and Sensory Evaluation. J. Food Sci..

[171] Hunt M.C., Kropf D.H., Pearson A.M. (1987). Color and appearance. Restructured Meat and Poultry Products, Advanced in Meat Research.

[172] Das A.K., Nanda P.K., Das A., Biswas S. (2019). Hazards and safety issues of meat and meat products. Food Safety and Human Health.

[173] Sharma A.K., Jana A.M., Srivastav A., Gupta M., Sharma S., Gill S.S. (2014). Antimicrobial properties of some edible mushrooms: A review. World J. Pharm. Pharm. Sci..

[174] Alves M., Ferreira I., Dias J., Teixeira V., Martins A., Pintado M. (2013). A review on antifungal activity of mushroom (*Basidiomycetes*) extracts and isolated compounds. Curr. Top. Med. Chem..

[175] Akyuz M., Onganer A.N., Erecevit P., Kirbag S. (2010). Antimicrobial activity of some edible mushrooms in the eastern and southeast anatolia region of Turkey. Gazi Univ. J. Sci..

[176] Lindequist U., Niedermeyer T.H.J., Jülich W.D. (2005). The pharmacological potential of mushrooms. Evid. Based Complement. Altern. Med..

[177] Hatvani N. (2001). Antibacterial effect of the culture fluid of Lentinus edodes mycelium grown in submerged liquid culture. Int. J. Antimicrob. Agents.

[178] Kitzberger C.S.G., Smânia A., Pedrosa R.C., Ferreira S.R.S. (2007). Antioxidant and antimicrobial activities of shiitake (*Lentinula edodes*) extracts obtained by organic solvents and supercritical fluids. J. Food Eng..

[179] Menaga D., Mahalingam P.U., Rajakumar S., Ayyasamy P.M. (2012). Evaluation of phytochemical characteristics and antimicrobial activity of Pleurotus florida mushroom. Asian J. Pharm. Clin. Res..

[180] Stojković D.S., Reis F.S., Ćirić A., Barros L., Glamočlija J., Ferreira I.C.F.R., Soković M. (2015). Boletus aereus growing wild in Serbia: Chemical profile, in vitro biological activities, inactivation and growth control of food-poisoning bacteria in meat. J. Food Sci. Technol..

[181] Das A.K., Nanda P.K., Bandyopadhyay S., Banerjee R., Biswas S., McClements D.J. (2020). Application of nanoemulsion-based approaches for improving the quality and safety of muscle foods: A comprehensive review. Compr. Rev. Food Sci. Food Saf..

[182] Yang J.H., Lin H.C., Mau J.L. (2001). Non-volatile taste components of several commercial mushrooms. Food Chem..

[183] Jabłońska-Ryś E., Skrzypczak K., Sławińska A., Radzki W., Gustaw W. (2019). Lactic Acid Fermentation of Edible Mushrooms: Tradition, Technology, Current State of Research: A Review. Compr. Rev. Food Sci. Food Saf..

